# Supplementary material for: Self-propelling vesicles define glycolysis as the minimal energy machinery for neuronal transport
Source: Nat Commun. 2016 Oct 24;7:13233. doi: 10.1038/ncomms13233 (PMC5078996; doi:10.1038/ncomms13233)
Supplement: Supplementary Information — Supplementary Figures 1-6. [file ncomms13233-s1.pdf]

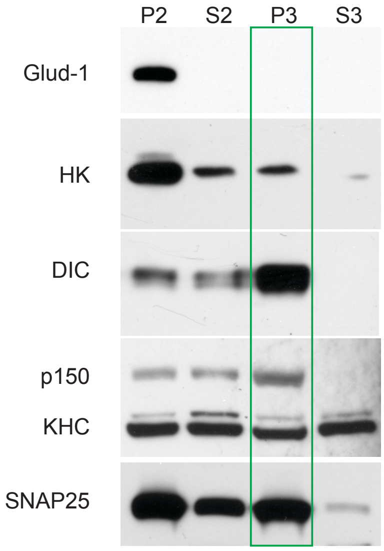


**Supplementary Figure 1: Vesicular fraction does not contain mitochondria.** Immunoblotting experiments of subcellular fractions separating large membranes (P2) from cytoplasm (S2), cytosol (S3) and small vesicles (P3). The mitochondrial matrix resident Glud1 is only present in the P2 fraction, known to be enriched in mitochondrial components and large membranes whereas glycolytic enzymes (HK) molecular motors (DIC, KHC), p150 Dynactin and SNAP25 are also present and enriched in the P3 fraction

a

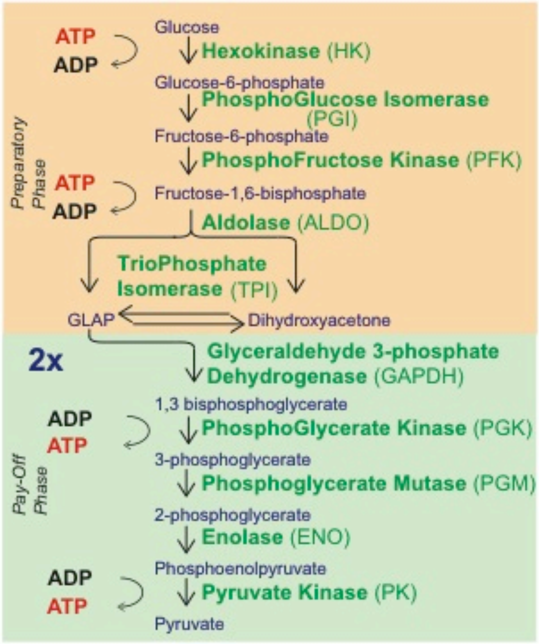


b

| | MS | WB | SNAP25 | Syn | VAMP2 mCh | Chrg A | BDNF mCh | APP mCh | FAT | ATP |
|---|---|---|---|---|---|---|---|---|---|---|
| HK | + | + | + | - | ND | ND | ND | ND | + | + |
| PGI | + | - | NA | NA | NA | NA | NA | NA | NA | ND |
| PFK | + | + | + | + | ND | ND | ND | ND | NA | ND |
| ALDO | + | + | + | + | + | + | + | + | NA | ND |
| TPI | + | NA | NA | NA | NA | NA | NA | NA | ND | ND |
| GAPDH | + | + | + | + | ND | ND | ND | ND | + | + |
| PGK | + | + | + | + | + | + | + | + | + | + |
| PGM | + | + | + | + | + | + | + | + | + | + |
| ENO | + | + | + | + | ND | ND | ND | ND | + | + |
| PK | + | + | + | + | + | + | - | + | + | + |

**Supplementary Figure 2: Glycolysis on vesicles.** (**a**) Schematic representation of the different steps of glycolysis. (**b**) The table on the right recapitulates the positive (+) or negative (-) outcome of the ten glycolytic enzymes in the different experimental paradigms. Note that all enzymes of the pay-off phase of the glycolysis are positive for each paradigm (NA: data not available due to technical issues, ND: not determined, MS: mass spectrometry, WB: Immunoprecipitation followed by Western blotting analysis, SNAP25 was analyzed by super-resolution microscopy, Synaptophysin, VAMP2-mCherry, Chromogranin A, BDNF-mCherry and APP-mCherry were analyzed by Airyscan confocal microscopy. FAT: Fast axonal transport, ATP: *in vitro* ATP production).

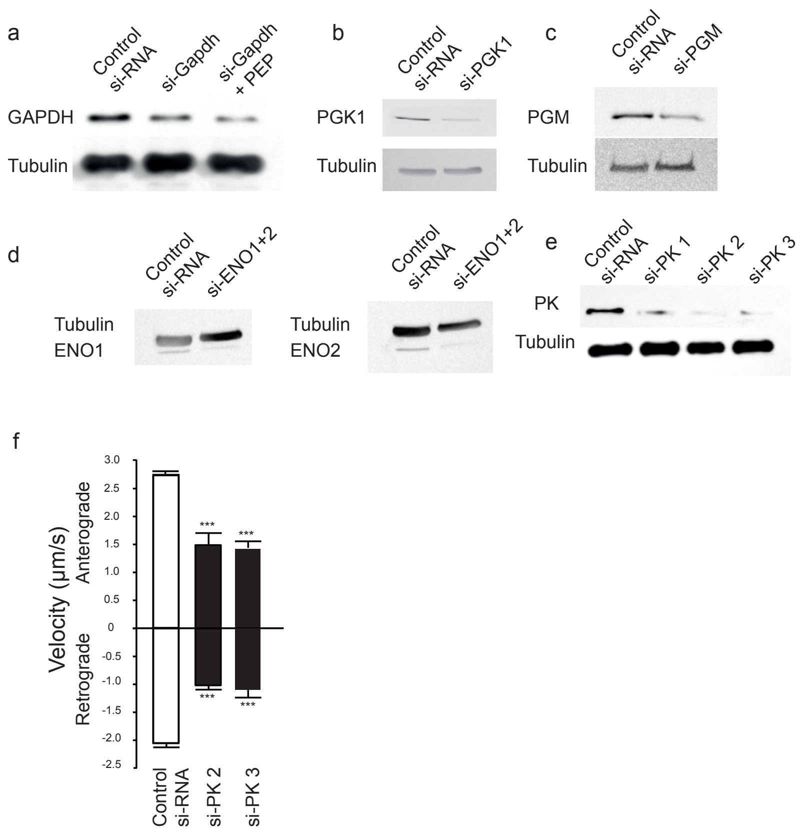


**Supplementary Figure 3: Silencing glycolytic enzymes reduces fast axonal transport in neurons.** (**a-e**) Silencing of GAPDH, PGK1, PGM, ENO1+2 and PK efficiently reduces protein expression. Immunoblotting experiments were done in parallel to each time lapse videomicroscopy experiment. (**f**) Two additional independent si-RNA targeting PK efficiently reduce FAT in neurons (anterograde velocity, F(2,866)59.5, $p=2.4 \times 10^{-10}$, control: n=473, si-PK2: n= 93, si-PK3: n=339; retrograde velocity, F(2.964), $p=7.1 \times 10^{-15}$, control: n=576, si-PK2: n=87, si-PK3: n=304) *** = p<0.001).

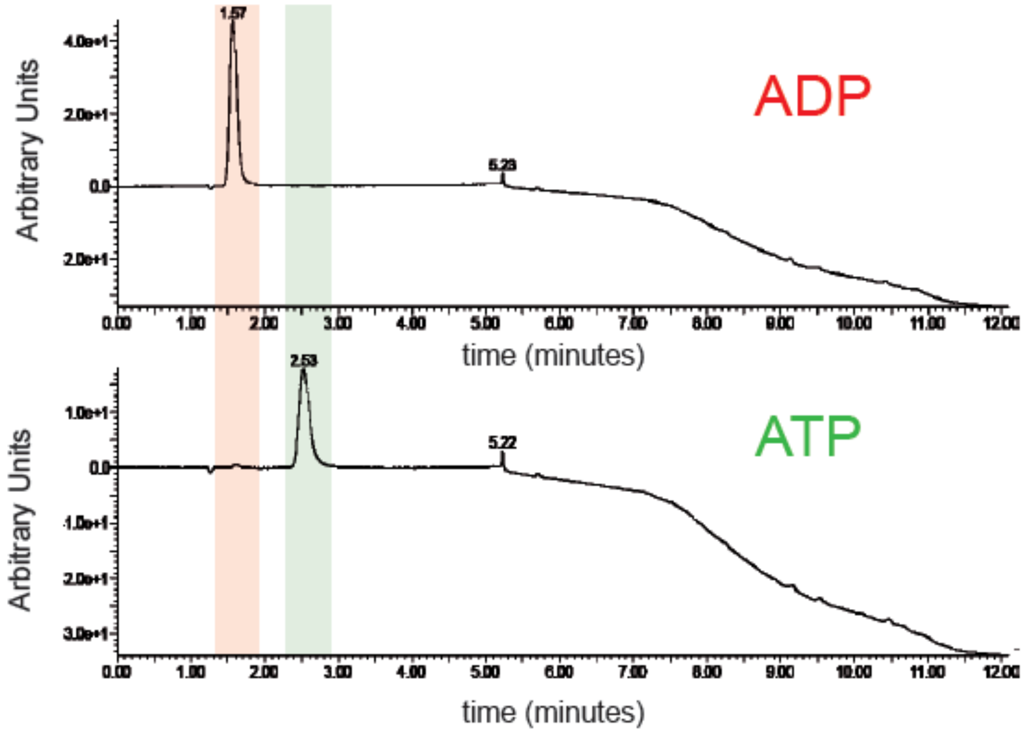


**Supplementary Figure 4: Analysis of ATP and ADP purity for *in vitro* motility assay.** The two graphs show the HPLC profile of 1 mM of ADP or 1 mM of ATP; the retention time is expressed in minutes and the intensity in arbitrary units (UA). The two products are characterized by a retention time of 1.57 minutes for ADP and 2.53 minutes for ATP. The purity of ADP is shown by the absence of the ATP peak at 2.53 minutes.

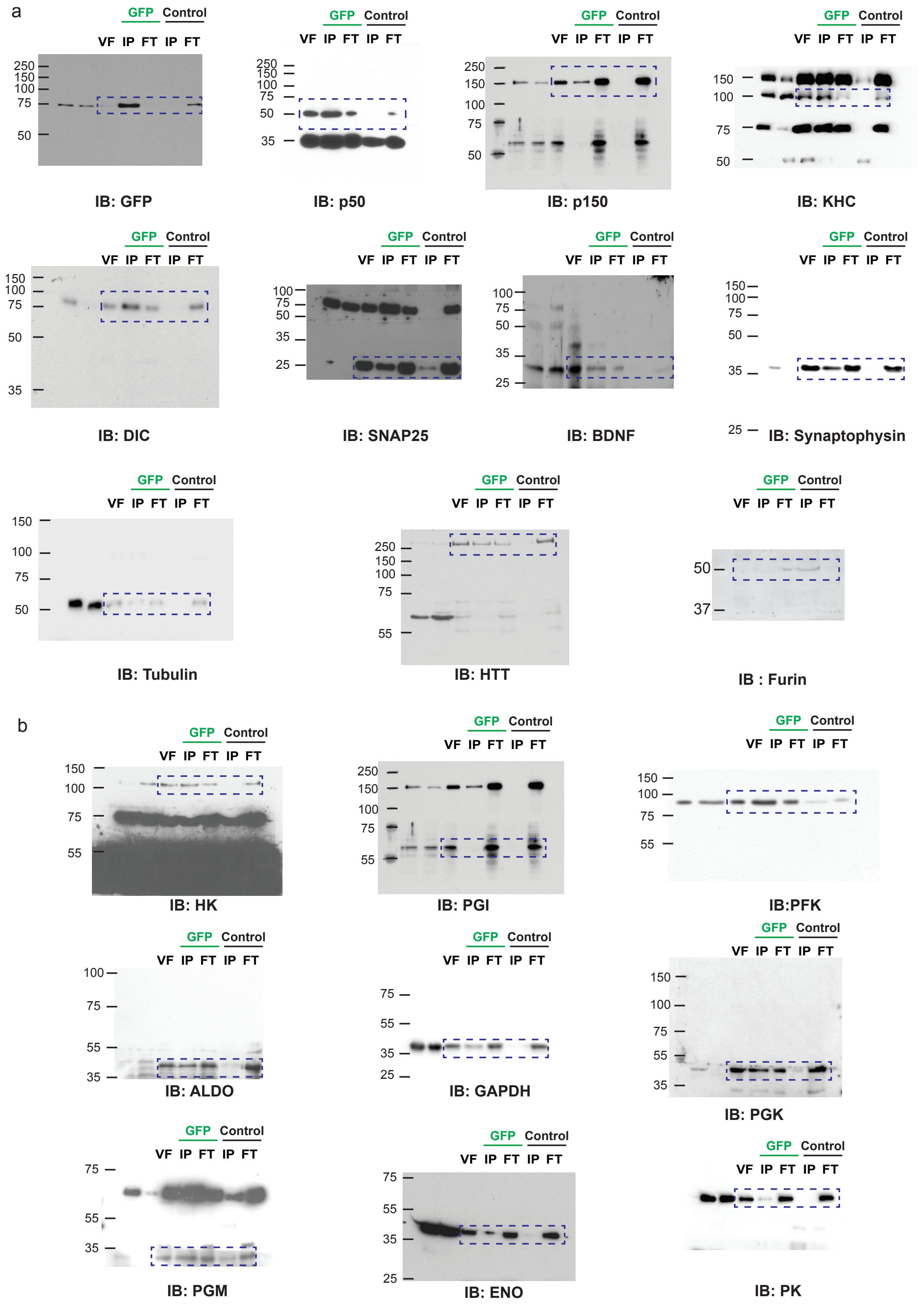


**Supplementary Figure 5: Images of uncropped immunoblots shown in the main text.** Images show the original immunoblots used for Figure 1b (a) and Figure 2b (b). Boxes indicate cropped regions and molecular weights are shown on the left of each blot. The antibodies used are indicated under each image. Extra bands observed correspond to incubation of the membrane with other antibodies.

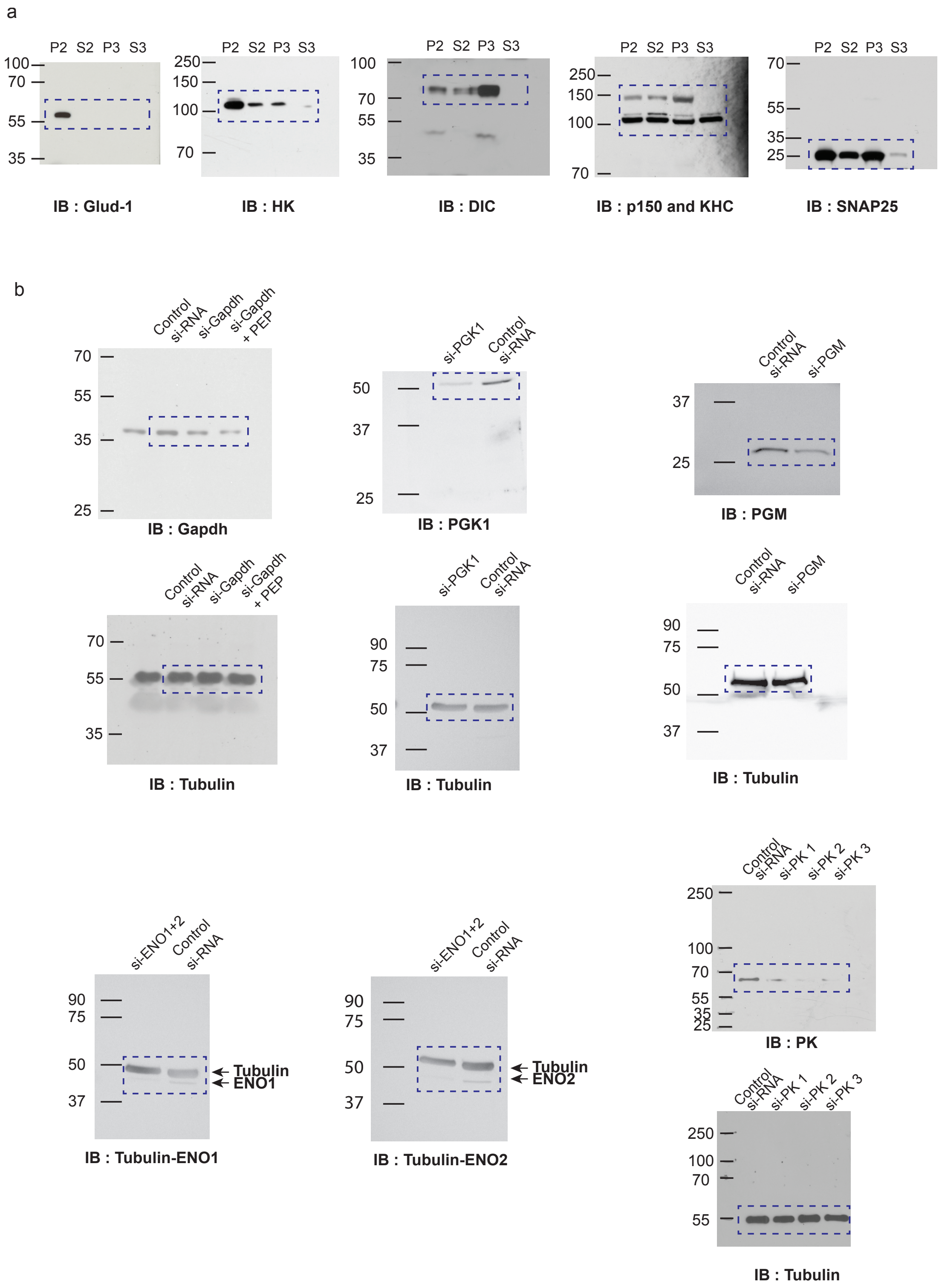


**Supplementary Figure 6: Images of uncropped immunoblots shown in the Supplementary Figures.** Images show the original immunoblots used for Supplementary Figure 1 (a) and Supplementary Figure 3 (b). Boxes indicate cropped regions and molecular weights are shown on the left of each blot. The antibodies used are indicated under each image.
